# Supplementary material for: From sequence to enzyme mechanism using multi-label machine learning
Source: BMC Bioinformatics. 2014 May 19;15:150. doi: 10.1186/1471-2105-15-150 (PMC4229970; doi:10.1186/1471-2105-15-150)
Supplement: Additional file 2 — Java code of ml2db. Additional file ml2db_code.tar.gz contains the Java source code to run the multi-label machine learning experiments and save the results to database. The code’s Javadoc is included. [file 1471-2105-15-150-S2.zip › additional file 2/ml2db/utils/doc/cern/jet/random/engine/MersenneTwister.html]

MersenneTwister


JavaScript is disabled on your browser.


- Overview
- Package
- Class
- Use
- Tree
- Deprecated
- Index
- Help

- Prev Class
- Next Class

- Frames
- No Frames

- All Classes

- Summary:
- Nested |
- Field |
- Constr |
- Method

- Detail:
- Field |
- Constr |
- Method


cern.jet.random.engine

## Class MersenneTwister

- java.lang.Object
- - cern.colt.PersistentObject
  - - edu.cornell.lassp.houle.RngPack.RandomElement
    - - edu.cornell.lassp.houle.RngPack.RandomSeedable
      - - cern.jet.random.engine.RandomEngine
        - - cern.jet.random.engine.MersenneTwister

- All Implemented Interfaces:
  :   DoubleFunction, IntFunction, java.io.Serializable, java.lang.Cloneable

  ---

    

  ```
  public class MersenneTwister
  extends RandomEngine
  ```

  MersenneTwister (MT19937) is one of the strongest uniform pseudo-random
  number generators known so far; at the same time it is quick. Produces
  uniformly distributed int's and long's in the closed
  intervals [Integer.MIN\_VALUE,Integer.MAX\_VALUE] and
  [Long.MIN\_VALUE,Long.MAX\_VALUE], respectively, as well as
  float's and double's in the open unit intervals
  (0.0f,1.0f) and (0.0,1.0), respectively. The seed can be
  any 32-bit integer except 0. Shawn J. Cokus commented that perhaps
  the seed should preferably be odd.

  **Quality:** MersenneTwister is designed to pass the k-distribution test.
  It has an astronomically large period of 2^19937^-1
  (=10^6001^) and 623-dimensional equidistribution up to 32-bit
  accuracy. It passes many stringent statistical tests, including the diehard test of G. Marsaglia
  and the load test of P. Hellekalek and S. Wegenkittl.

  **Performance:** Its speed is comparable to other modern generators (in
  particular, as fast as java.util.Random.nextFloat()). 2.5 million
  calls to raw() per second (Pentium Pro 200 Mhz, JDK 1.2, NT). Be
  aware, however, that there is a non-negligible amount of overhead required to
  initialize the data structures used by a MersenneTwister. Code like

  ```
   double sum = 0.0;
   for (int i = 0; i < 100000; ++i) {
          RandomElement twister = new MersenneTwister(new java.util.Date());
          sum += twister.raw();
   }
  ```

  will be wildly inefficient. Consider using

  ```
   double sum = 0.0;
   RandomElement twister = new MersenneTwister(new java.util.Date());
   for (int i = 0; i < 100000; ++i) {
          sum += twister.raw();
   }
  ```

  instead. This allows the cost of constructing the MersenneTwister object to
  be borne only once, rather than once for each iteration in the loop.

  **Implementation:** After M. Matsumoto and T. Nishimura,
  "Mersenne Twister: A 623-Dimensionally Equidistributed Uniform Pseudo-Random Number Generator"
  , ACM Transactions on Modeling and Computer Simulation, Vol. 8, No. 1,
  January 1998, pp 3--30.

  More info on Masumoto's homepage. More info on Pseudo-random number generators is on the Web. Yet some more info. The correctness of this implementation has been verified against the published output sequence mt19937-2.out of the C-implementation mt19937-2.c. (Call test(1000) to print the sequence). Note that this implementation is **not synchronized**. **Details:** MersenneTwister is designed with consideration of the flaws of various existing generators in mind. It is an improved version of TT800, a very successful generator. MersenneTwister is based on linear recurrences modulo 2. Such generators are very fast, have extremely long periods, and appear quite robust. MersenneTwister produces 32-bit numbers, and every k-dimensional vector of such numbers appears the same number of times as k successive values over the period length, for each k <= 623 (except for the zero vector, which appears one time less). If one looks at only the first n <= 16 bits of each number, then the property holds for even larger k, as shown in the following table (taken from the publication cited above): | | | | | | | | | | | | | | | | --- | --- | --- | --- | --- | --- | --- | --- | --- | --- | --- | --- | --- | --- | | n | 1 | 2 | 3 | 4 | 5 | 6 | 7 | 8 | 9 | 10 | 11 | 12 .. 16 | 17 .. 32 | | k | 19937 | 9968 | 6240 | 4984 | 3738 | 3115 | 2493 | 2492 | 1869 | 1869 | 1248 | 1246 | 623 | MersenneTwister generates random numbers in batches of 624 numbers at a time, so the caching and pipelining of modern systems is exploited. The generator is implemented to generate the output by using the fastest arithmetic operations only: 32-bit additions and bit operations (no division, no multiplication, no mod). These operations generate sequences of 32 random bits (int's). long's are formed by concatenating two 32 bit int's. float's are formed by dividing the interval [0.0,1.0] into 2^32^ sub intervals, then randomly choosing one subinterval. double's are formed by dividing the interval [0.0,1.0] into 2^64^ sub intervals, then randomly choosing one subinterval.

  Version:
  :   1.0, 09/24/99

  Author:
  :   wolfgang.hoschek@cern.ch

  See Also:
  :   `Ranmar`,
      `Ranlux`,
      `Ranecu`,
      `Random`,
      Serialized Form

- - ### Field Summary

    Fields

    | Modifier and Type | Field and Description |
    | --- | --- |
    | `static int` | `DEFAULT_SEED` |
  - ### Constructor Summary

    Constructors

    Constructor and Description

    `MersenneTwister()`

    Constructs and returns a random number generator with a default seed,
    which is a **constant**.

    `MersenneTwister(java.util.Date d)`

    Constructs and returns a random number generator seeded with the given
    date.

    `MersenneTwister(int seed)`

    Constructs and returns a random number generator with the given seed.
  - ### Method Summary

    Methods

    | Modifier and Type | Method and Description |
    | --- | --- |
    | `java.lang.Object` | `clone()` Returns a copy of the receiver; the copy will produce identical sequences. |
    | `int` | `nextInt()` Returns a 32 bit uniformly distributed random number in the closed interval [Integer.MIN\_VALUE,Integer.MAX\_VALUE] (including Integer.MIN\_VALUE and Integer.MAX\_VALUE). |

    - ### Methods inherited from class cern.jet.random.engine.RandomEngine

      `apply, apply, makeDefault, nextDouble, nextFloat, nextLong, raw`
    - ### Methods inherited from class edu.cornell.lassp.houle.RngPack.RandomSeedable

      `ClockSeed, ClockSeed`
    - ### Methods inherited from class edu.cornell.lassp.houle.RngPack.RandomElement

      `choose, choose, gaussian, gaussian, powlaw, raw, raw, uniform`
    - ### Methods inherited from class java.lang.Object

      `equals, getClass, hashCode, notify, notifyAll, toString, wait, wait, wait`

- - ### Field Detail


    - #### DEFAULT\_SEED

      ```
      public static final int DEFAULT_SEED
      ```

      See Also:
      :   Constant Field Values
  - ### Constructor Detail


    - #### MersenneTwister

      ```
      public MersenneTwister()
      ```

      Constructs and returns a random number generator with a default seed,
      which is a **constant**. Thus using this constructor will yield
      generators that always produce exactly the same sequence. This method is
      mainly intended to ease testing and debugging.


    - #### MersenneTwister

      ```
      public MersenneTwister(java.util.Date d)
      ```

      Constructs and returns a random number generator seeded with the given
      date.

      Parameters:
      :   `d` - typically new java.util.Date()


    - #### MersenneTwister

      ```
      public MersenneTwister(int seed)
      ```

      Constructs and returns a random number generator with the given seed.

      Parameters:
      :   `seed` - should not be 0, in such a case
          MersenneTwister.DEFAULT\_SEED is silently substituted.
  - ### Method Detail


    - #### clone

      ```
      public java.lang.Object clone()
      ```

      Returns a copy of the receiver; the copy will produce identical
      sequences. After this call has returned, the copy and the receiver have
      equal but separate state.

      **Overrides:**
      :   `clone` in class `PersistentObject`

      Returns:
      :   a copy of the receiver.


    - #### nextInt

      ```
      public int nextInt()
      ```

      Returns a 32 bit uniformly distributed random number in the closed
      interval [Integer.MIN\_VALUE,Integer.MAX\_VALUE] (including
      Integer.MIN\_VALUE and Integer.MAX\_VALUE).

      **Specified by:**
      :   `nextInt` in class `RandomEngine`


- Overview
- Package
- Class
- Use
- Tree
- Deprecated
- Index
- Help

- Prev Class
- Next Class

- Frames
- No Frames

- All Classes

- Summary:
- Nested |
- Field |
- Constr |
- Method

- Detail:
- Field |
- Constr |
- Method
